# Epigenetic Upregulation of Carotid Body Angiotensin Signaling Increases Blood Pressure

**DOI:** 10.1161/HYPERTENSIONAHA.124.23349

**Published:** 2024-12-05

**Authors:** Fengli Zhu, Zhuqing Wang, Kayla Davis, Hayden McSwiggin, Jekaterina Zyuzin, Jie Liu, Wei Yan, Virender K. Rehan, Nicholas Jendzjowsky

**Affiliations:** 1 The Lundquist Institute for Biomedical Innovation (F.Z., Z.W., K.D., H.M., J.Z., J.L., W.Y., V.K.R., N.J.), Harbor-UCLA Medical Center, Torrance.; 2 Division of Metabolic Diseases and Translational Genomics (W.Y.), Harbor-UCLA Medical Center, Torrance.; 3 Division of Neonatology (V.K.R.), Harbor-UCLA Medical Center, Torrance.; 4 Division of Respiratory and Critical Care Medicine and Physiology (N.J.), Harbor-UCLA Medical Center, Torrance.; 5 David Geffen School of Medicine, University of California, Los Angeles (W.Y., V.K.R., N.J.).

**Keywords:** angiotensin, carotid body, epigenomics, hypertension, nicotine

## Abstract

**BACKGROUND:**

Epigenetic changes can be shaped by a wide array of environmental cues, maternal health, and behaviors. One of the most detrimental behaviors to the developing fetus is nicotine exposure. Perinatal nicotine exposure remains a significant risk factor for cardiovascular health and, in particular, hypertension. Increased basal carotid body (CB) activity and excitation are significant contributors to hypertension. This study investigated the epigenetic changes to CB activity induced by perinatal nicotine exposure resulting in CB-mediated hypertension.

**METHODS:**

We used a rodent model of perinatal nicotine exposure and cell culture methods.

**RESULTS:**

We show that the AgtR1 (angiotensin II type 1 receptor) is upregulated in the carotid bodies of nicotine-exposed offspring. These changes were attributed to an upregulation of genetic promotion as DNA methylation of AgtR1 occurred within intron regions, exemplifying an upregulation of genetic transcription for this gene. Nicotine increased angiotensin signaling in vitro. CB reactivity to angiotensin was increased in perinatal nicotine-exposed offspring compared with control offspring. Furthermore, CB denervation reduced arterial pressure because of suppressed efferent sympathetic activity in perinatal nicotine-exposed offspring.

**CONCLUSIONS:**

Our data demonstrate that perinatal nicotine exposure adversely affects CB afferent sensing, which augments efferent sympathetic activity to increase vasoconstrictor signaling and induce hypertension. Targeting angiotensin signaling in the carotid bodies may provide a way to alleviate hypertension acquired by adverse maternal uterine environments in general and perinatal nicotine exposure in particular.

**NOVELTY AND RELEVANCE**

**What Is New?**

- Adverse maternal programming in the form of perinatal nicotine exposure can produce epigenetic modifications to augment AgtR1 (angiotensin II type 1 receptor) signaling in the carotid body.

**What Is Relevant?**

- Increased epigenetic changes, which can augment the carotid body’s basal signaling or excitation state, will increase efferent sympathetic activity to augment blood pressure.

**Clinical/Pathophysiological Implications?**

- Maternal/perinatal history could profoundly impact the child’s cardiovascular health by increasing neurogenic signaling to increase blood pressure. Neurogenic hypertension has been tied to drug-resistant hypertension.

Adverse developmental programming is directly related to cardiovascular disease in later life. The epigenetic modifications that occur in response to intrauterine alterations have lasting effects and are known to alter cardiovascular structure and function in response to several environmental factors and maternal behaviors.^1–5^ One of the most detrimental maternal behaviors during the perinatal period, which deleteriously predisposes offspring to cardiovascular diseases, is nicotine exposure, typically in the form of tobacco product use.^6^

Perinatal nicotine exposure has been shown to augment blood pressure and increase the risk of hypertension in later life.^2,6,7^ Arteriolar wall thickening and increased vasoconstrictor signaling are thought to be the primary cause of nicotine exposure–induced hypertension.^8–10^ Nicotine stimulates the renin-angiotensin-aldosterone system^11^ and increases both angiotensin-converting enzyme activity,^12,13^ as well as AgtR1 (angiotensin II type 1 receptor) expression and activity^12–14^ within the aorta^15–17^ and mesenteric arterioles.^16,18^ Such arteriolar reactivity would likely implement a hypertensive state.

Aberrant sympathetic nerve activity is significantly tied to cardiovascular disease and a prime mediator of hypertension in a multitude of cardiometabolic diseases.^19,20^ Carotid bodies are polymodal sensors that regulate sympathetic nervous activity to control vasoconstriction to regulate tissue oxygen/nutrient delivery.^21,22^ Indeed, hypertension critically depends on tonic input from carotid bodies to augment sympathetic tone.^19,20^ Carotid body (CB) activity of nicotine-exposed offspring is not suppressed by hyperoxia, which typically quiesces CB activity, suggesting an augmented basal sensitization.^23,24^ Given their primary role in stimulating arteriolar vasoconstriction^19,20^ and their susceptibility to nicotine exposure,^23,24^ we reasoned that the carotid bodies are likely susceptible to epigenetic modulation because of developmental programming.^25–27^ Therefore, we investigated the hypothesis that carotid bodies are susceptible to epigenetic hypersensitization, which increases blood pressure in perinatal nicotine-exposed offspring.

## Methods

Additional methodological details may be found in the Supplemental Material.

### Data Availability

RNA sequencing and DNA bisulfite sequencing data can be found in the National Institutes of Health Sequence Read Archive (Bioproject ID: PRJNA1106547). All other data are found in the supporting data and Supplemental Material and can be further made available upon request to the corresponding author.

### Animal Models, Care, and Ethical Standards

First-time pregnant Sprague-Dawley rat dams were purchased from Charles River (Hollister, CA) at gestational day 3. Mothers were subject to subcutaneous nicotine injections (2 mg/kg, MilliporeSigma, PHR2532) from gestational day 7 to postnatal day (PND) 21.^28–30^ Control mothers received phosphate-buffered salive (vehicle). Animals were housed with controlled temperature (21 °C ±2 °C), humidity (55±10%), and 12 hours light-dark cycle. Pups were weaned on PND 21 and housed in pairs. Animals had unlimited access to food and water. Experiments took place from PNDs 56 to 80. All experimental procedures were approved by The Lundquist Institute for Biomedical Innovation at Harbor-UCLA Medical Center IACUC board (protocol No. 31659).

Assignment of control or nicotine to mothers was block-randomized to minimize experimenter bias. In vitro and in vivo power calculations were based on an estimated effect size of 50% difference with assumed unified SD across groups.

### PC12 Culture

Cells of rat adrenal pheochromocytoma (PC12) were cultured in RPMI 1640 (Gibco; No. 11875093) supplemented with 10% Horse Serum (Gibco; Cat. No. 26050-088), 5% fetal bovine serum (Gibco; Cat. No. F2442), 0.4-µg/mL dexamethasone (MilliporeSigma; Cat. No. D4902), and 1% penicillin/streptomycin (MilliporeSigma; Cat. No. P0781).

### Next-Generation RNA Sequencing

On PND 56, animals were sedated (5% isoflurane, balance O_2_) and exsanguinated, and carotid bodies were prepared for RNA isolation. Five CB pairs (n=10 carotid bodies from 5 rats) from each group and sex (n=5 rats per group, per sex) were lysed, and RNA was isolated using the easy-spin Total RNA extraction kit (Boca Scientific; Cat. No. 17221). RNA sequencing was then conducted by an unbiased commercial vendor (Novogene Corporation, Sacramento, CA).

### Whole Genome DNA-Bisulfite Sequencing

Carotid bodies were dissected as above. Five CB pairs (n=10 carotid bodies from 5 rats) from each group and sex (n=5 rats per group, per sex) were lysed, and DNA was isolated using the QIAamp UCP DNA Micro Kit (Qiagen; Cat. No. 56204). Bisulfite conversion and downstream whole-genome DNA-bisulfite sequencing^31–37^ were conducted by (Novogene Corporation).

### RNA Quantitative Polymerase Chain Reaction of Carotid Bodies

Quantitative polymerase chain reaction was conducted with TaqMan specific probes: AgtR1a (angiotensin II type 1a receptor; Thermo Fisher; Rn02758772_s1), AgtR2 (angiotensin II type 2 receptor; Thermo Fisher; Rn00560677_s1), angiotensinogen (Thermo Fisher; RN00593114_m1), angiotensin-converting enzyme (Thermo Fisher; Rn00561094_m1), renin (Thermo Fisher; Rn02586313_m1), tyrosine hydroxylase (Thermo Fisher Rn00562500_m1), EPAS1 (endothelial PAS domain-containing protein 1)/Hif2α (hypoxia-inducible factor 2 alpha; Rn00576515_m1), and TRPV1 (transient receptor potential vanilloid 1 channel; Thermo Fisher; Rn00583117_m1). All experimental genes were evaluated in reference to HPRT (hypoxanthine-guanine phosphoribosyltransferase; Thermo Fisher; Rn01527840_m1). Delta CT was calculated in reference to HPRT, and comparisons between groups were made using an unpaired 2-sided *t* test.

### Methylated DNA Immunoprecipitation-Quantitative Polymerase Chain Reaction

Methylated DNA immunoprecipitation was performed using the methylated DNA immunoprecipitation kit (Cat. No. ab117133, Abcam) following the procedures described previously^38^ to identify DNA methylation levels. Primer sequence-AgtR1: forward: TACCTAAACATAGTAAAAGCCAAACACA; AgtR1: reverse: TATCCTGTTGATCTCTTTTTGTTGTCTG. The difference in cycle threshold (ΔCt) was calculated as methylated AgtR1 DNA-total AgtR1 DNA and was used to plot the data.^38,39^ Comparisons between groups were made using an unpaired 2-sided *t* test.

### Immunoblot of PC12 Cells

After incubation with or without nicotine, PC12 cells were lysed and stored at −80 °C until analysis. The cell lysate was loaded onto bis-tris gels and transferred onto polyvinylidene fluoride membranes. Membranes were incubated with actin primary antibody (Sigma; Cat. No. A5441-2 mL, 1:5000) and goat-anti-mouse antibody (Invitrogen; Cat. No. 31430, 1:5000). Blots were stripped with horseradish peroxidase stripping buffer (Azure Biosystems Cat. No. AC2154) and reincubated with AgtR1 primary antibody (Proteintech; Cat. No. 25343-1-AP, 1:600) and goat anti-rabbit antibody (Invitrogen; Cat. No. 31460, 1:5000). Bands were quantified with Biorad ImageLab, and the AgtR1 signal was normalized to actin. Groups were compared using an unpaired 2-sided *t* test.

### Immunohistochemistry

Carotid bodies were grossly dissected as previously.^40^ Tissues were fixed in 4% paraformaldehyde for 1 to 2 hours at 4 °C and then cryopreserved in 30% sucrose overnight at 4 °C, and 14-µm sections were cut with a cryostat. Sections were incubated with antibodies for TH (tyrosine hydroxylase; MilliporeSigma; ZMS1033, clone 20/40/15), AgtR1 (MilliporeSigma; AB15552, polyclonal), and TRPV1 (Alomone Labs; ACC-030, polyconal) and subsequently incubated with appropriate secondaries (Jackson Laboratories, goat-anti-rabbit Cy3 111-165-003, Cy5 111-005-003, and goat-anti-mouse 488 115-545-003), counterstained with 4′,6-diamidino-2-phenylindole (MilliporeSigma; MBD0015), and coverslipped with prolong diamond antifade mounting media (Thermo Fisher; No. P36970). Slides were imaged using the Leica Thunder imaging acquisition system.

### Calcium Imaging

PC12 cells were separated into media (control), nicotine (Sigma; Cat. No. 6019-06-3, 50 µg/mL), Ang II (angiotensin II; Tocris; Cat. No. 1158, 5 µM), nicotine+Ang II, Ang II+losartan (Tocris; Cat. No. 3798, 3 µM), nicotine+Ang II+losartan, Ang II+AMG9810 (TRPV1 antagonist; Tocris, Cat. No. 2316, 10 µM), nicotine+Ang II+AMG9810, or nicotine+Ang II+losartan+AMG9810. Cells were then treated with Calcium Orange acetoxymethylmethyl ester (Invitrogen; Cat. No. C3015)+0.01% pluronic acid (Invitrogen; Cat. No. F-127). Cells were imaged in the Incucyte imaging system (Sartorius). The frequency of calcium bursts was calculated by Incucyte software and averaged for each well to express a single value. Data were analyzed with 1-way ANOVA using the Holm-Sidak post hoc test.

### In Vivo CB Reactivity

Rats were anesthetized with 5% isoflurane (balance room air) using the Kent Scientific Somnosuite anesthesia system. The femoral artery and vein were cannulated, and the arterial line was instrumented to a pressure transducer to measure continuous arterial pressure (ADInstruments MLT0670) using the PowerLab data acquisition console (ADInstruments PL3516/P). The vein was then connected to a syringe pump (Fisher Scientific; Cat. No. 14831200) to induce a 15-mg/kg/min infusion of alfaxan (Victor Medical; Cat. No. 1629020).^41,42^ Then, the jugular vein was cannulated to deliver bolus injections of Ang II (Tocris; Cat. No. 1158, 1 mg/kg) and sodium cyanide (Fisher Scientific; Cat. No. AA1213722, 200 µg/kg). These injections were repeated following bilateral CB denervation. Mean arterial pressure was averaged at the 60s preceding each bolus injection and a 60s average during the peak reactive response to each bolus injection. Data were analyzed by 2-way ANOVA (group×CB intact versus denervated) for Ang II or sodium cyanide injection. The Holm-Sidak post hoc test determined group differences.

### Serum Angiotensin Concentration

Serum was drawn, aliquoted, snap-frozen, and stored at −80 °C until analysis. Angiotensin enzyme-linked immunosorbent assay (RayBiotech; No. EIAR-ANGII-1) was used per the manufacturer’s instructions. Groups (control pups versus perinatal nicotine-exposed pups) were analyzed with a 2-sided unpaired *t* test.

### Arterial Pressure Assessment Between Groups

Rats from control or perinatal nicotine-exposed mothers were assessed with a noninvasive tail plethysmography system (IITC Life Science) to assess blood pressure. Data were compared using an unpaired 2-sided *t* test.

### CB-Mediated Changes in Arterial Pressure

Rats were anesthetized with 5% isoflurane (balance O_2_) as induction and maintained on a surgical plane with 2% to 3% isoflurane. The Kaha telemetry dual sympathetic nerve activity/pressure (TRM56SP, ADInstruments) was instrumented around the renal nerves using KwikGard dental impression material to isolate electrodes from electrical interference and abdominal movements for renal sympathetic nerve activity (RSNA) recording. The pressure transducer was inserted into the aorta as per the manufacturer’s instructions utilizing Vetbond to measure continuous pressure measurement. Pressure and RSNA were recorded for 1 week; carotid sinus nerves were denervated. Buprenorphine was delivered as above, and recording commenced for another week following recovery. Arterial pressure waveforms delineated heart rate, systolic, diastolic, mean arterial pressure, pulse pressure, and respiratory rate. RSNA was filtered, rectified, and integrated using LabChart 8. Data were binned into daily averages to obtain 7 days of values, pre- and post-CB denervation, for each rat and analyzed with a prepost with a 2-sided paired *t* test.

### Statistics

Specific statistical tests are described for each experiment above. Data were averaged and analyzed for each experiment as described above. Data were analyzed using GraphPad, v10.

## Results

### Epigenetic Changes in the CB in Response to Perinatal Nicotine Exposure

**Figure 1.**
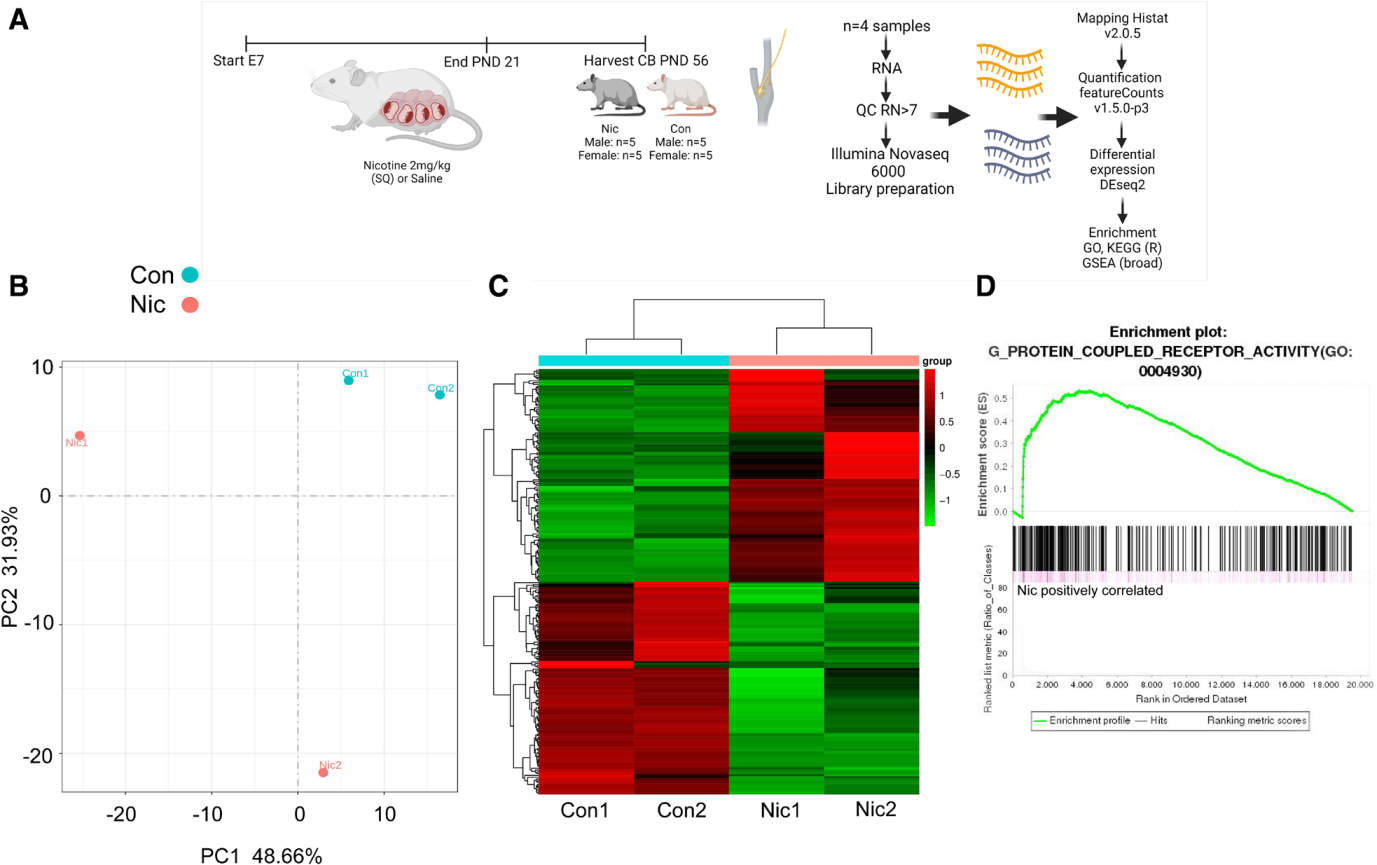
**Perinatal nicotine (Nic) exposure alters RNA transcripts associated with angiotensin signaling in the carotid body (CB). A**, Transcriptomic study design. Nic or saline was delivered to the mother on estrous day 7 (E7) to postnatal day (PND) 21. Bilateral CB samples were microdissected from perinatal Nic or saline (control [Con]) exposed rats on PND 56. (n=5 rats per group; tissues were pooled to yield sufficient RNA for analysis.) Two male and 2 female groups per condition. **B**, Principal component analysis (PCA) plot showing distinct separation between Nic and Con groups. PCA1 was group, and PCA2 was sex. **C**, Heatmap showing overall differences in RNA distribution. **D**, Gene set enrichment analysis (GSEA) plot for G-protein–coupled receptor activity (0004930) where AgtR1a (angiotensin II type 1a receptor) was significantly increased in Nic -exposed offspring compared with Con s. Technical replicates, n=2 per group (1 male, 1 female). Each n consists of 10 carotid bodies from 5 rats each. GO indicates gene ontology; and KEGG, Kyoto Encyclopedia of Genes and Genome Ontology Analysis.

To investigate possible genetic changes expounded by adverse maternal behaviors, we implemented a standardized model of perinatal nicotine exposure.^28–30^ We conducted an unbiased transcriptomic screen of carotid bodies harvested from perinatal nicotine-exposed pups on PND 56, 5 weeks after weaning, and the final dose of nicotine was delivered to the mother. We pooled together carotid bodies from 5 pups in each group of nicotine-exposed and saline-control pups (Figure 1A). Samples were analyzed by sex, but no sex difference was found. A total of 922 genes were differentially expressed between conditions, with 685 upregulated, 237 downregulated, and 19 196 unchanged transcripts (Figure 1B and 1C). AgtR1a was significantly upregulated in nicotine-exposed offspring compared with control (0.69 log2-fold change; *P*_adj_=0.04). AgtR2 was downregulated by perinatal nicotine exposure (log2-fold change, −3.246; *P*_adj_=6.65×10^−12^). No differences in angiotensinogen (log2-fold change, 0.233; *P*_adj_=0.693), angiotensin-converting enzyme (log 2-fold change, −0.071; *P*_adj_=0.871), or renin (log2-fold change, −2.248; *P*_adj_=0.645) were demonstrated (Data S1). Gene set enrichment analysis was performed on the differentially expressed gene, revealing upregulated gene ontology pathways, including gene ontology:0004930 (normalized enrichment score, 1.539; Figure 1D; Data S2), which included AgtR1a.

To validate these findings, we conducted immunohistochemistry and quantitative polymerase chain reaction to identify possible upregulation of potential signaling pathways in the carotid bodies between perinatal nicotine exposed and control pups. We show that AgtR1a was significantly upregulated in perinatal nicotine-exposed offspring compared with the control. AgtR2 and TRPV1 transcription did not significantly increase (Figure S1).

To test whether changes in angiotensin signaling occurred outside the carotid bodies, we tested whether RNA transcripts of AgtR1a, AgtR2, angiotensinogen, angiotensin-converting enzyme, or renin were altered in the kidney and brains of perinatal nicotine exposed pups compared with controls. We observed that angiotensinogen and angiotensin-converting enzymes were significantly upregulated in the brains and kidneys of perinatal nicotine-exposed rats compared with the control, and renin was downregulated in the kidneys of perinatal nicotine-exposed rats compared with the control with no change in the brain (Figure S2). In contrast, AgtR1a and AgtR2 were unchanged in the brains and kidneys of perinatal nicotine-exposed rats compared with control rats (Figure S2).

**Figure 2.**
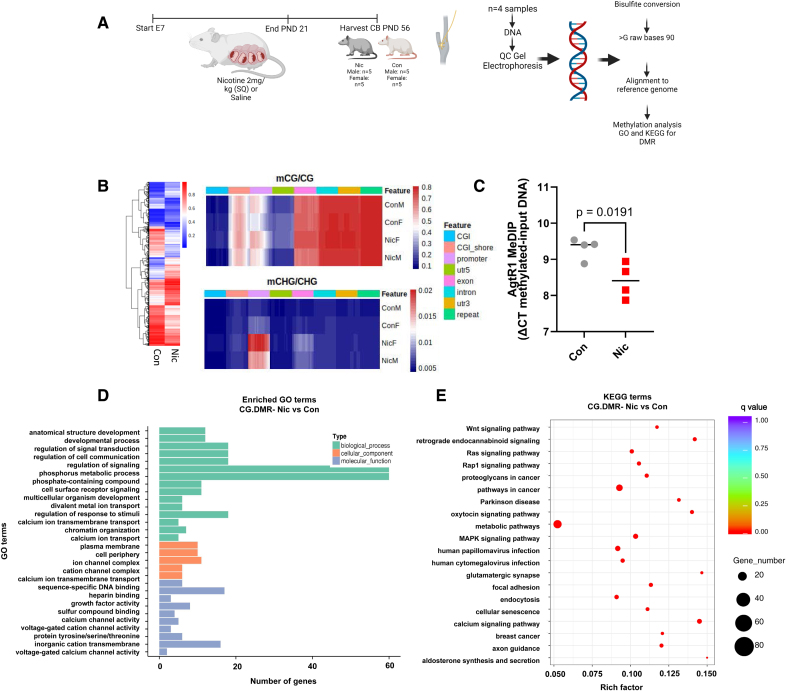
**Perinatal nicotine (Nic) exposure alters DNA methylation in the carotid body (CB). A**, DNA-methylation study design. Nic or saline was delivered to the mother on estrous day 7 (E7) to postnatal day (PND) 21. Bilateral CB samples were microdissected from perinatal Nic or saline (control [Con]) exposed rats on PND 56. n=1 male replicate and 1 female replicate per group where 1 replicate consists of 10 carotid bodies from 5 rats. One male and 1 female group per condition. **B**, Heatmap displaying cytosine-guanine (CG) and cytosine-arginine/threonine/guanine-guanine (CHG) methylation differences in carotid bodies between Nic and saline-exposed rats. The mapping of methylation changes is confined to the CG and CHG regions as per the heat maps. **C**, Immunoprecipitation of methylated DNA compared with whole DNA demonstrates an increase in methylated AgtR1 (angiotensin II type 1 receptor) DNA in the carotid bodies of Nic exposed compared with Con offspring. The difference in cycle threshold (ΔCt) was used to compare differences between groups (lower value equals greater amount of methylation). n=4 per group where each n consists of 10 carotid bodies from 5 rats. **D**, Enriched gene ontology (GO) terms for CG-methylated DNA between Nic-exposed and Con pups. **E**, Dot plot showing the gene number and enrichment factor of Kyoto Encyclopedia of Genes and Genome (KEGG) pathways for CG-methylated DNA between Nic-exposed and Con pups.

Given that our goal was to elucidate possible epigenetic differences responsible for mediating a neurogenic form of hypertension, we probed further and conducted whole genome DNA bisulfite sequencing^43^ of the carotid bodies from perinatal nicotine-exposed and control pups (Figure 2A). Samples were analyzed by sex, but no sex difference was found. Mapping the whole genome of the carotid bodies between groups revealed that the predominant changes to DNA methylation occurred in the promoter (suppression) and intron/exon regions (enhancement; Figure 2B). Methylation was distributed throughout the genome where a differential set of genes was altered on the cytosine-guanine and cytosine-arginine/threonine/guanine-guanine regions (Figure 2B). Gene ontology analyses of whole genes (Figure 2D) and promoter regions (Figure 2E) revealed similar gene ontology terms with transcriptomic changes. Importantly, a significant difference in methylation AgtR1a between groups was demonstrated with a difference of 0.448319 between perinatal nicotine exposed and control rats (Data S3). AgtR1 was orthogonally confirmed with immunoprecipitation of methylated DNA compared with total DNA amplifying AgtR1 to assess global methylation (Figure 2C). AgtR1 DNA was not significantly methylated in brains but was increased in the kidneys of perinatal nicotine-exposed rats compared with controls (Figure S3). Proteins for AgtR1, TRPV1, and TH (a standard marker of CB glomus cells) were all present in sections of carotid bodies harvested from perinatal nicotine-exposed offspring where AgtR1 is on glomus cells, and TRPV1 appears to oppose glomus cells on the postsynaptic terminals^44^ (Figure S1); however, our resolution is insufficient to determine the localization of glomus cells definitively. As such, our staining may not reflect the accurate location of AgtR1 in rat CB glomus cells.

### Nicotine Upregulates AgtR1 Activity

**Figure 3.**
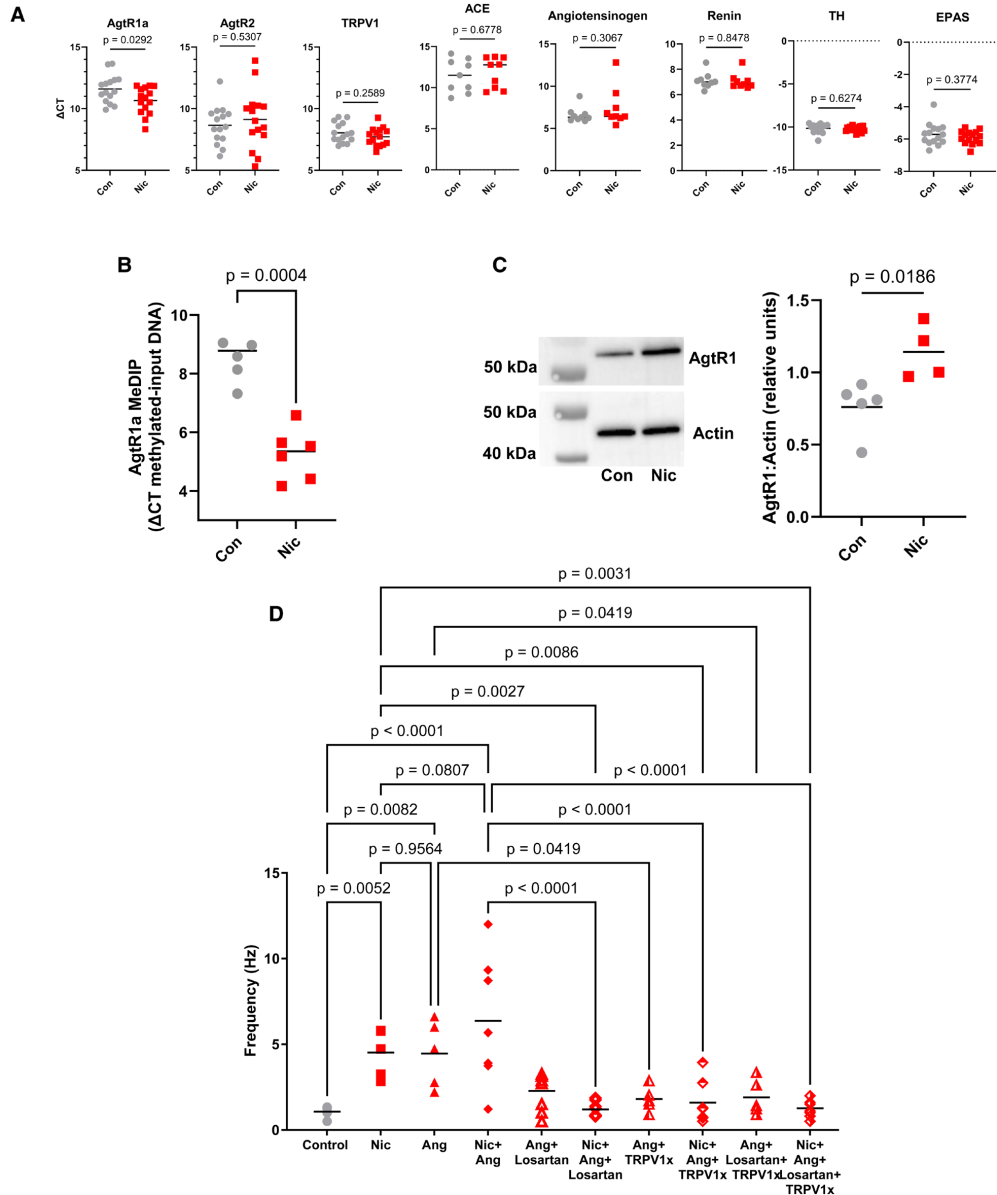
**Nicotine increases the AgtR1 (angiotensin II type 1 receptor) pathway in cultured cells of rat adrenal pheochromocytoma (PC12). A**, PC12 cells were cultured for 6 hours with and without 50-µg/mL nicotine (similar concentration to a single dose of nicotine used in vivo); AgtR1a (angiotensin II type 1a receptor) was increased by nicotine (T_28_=2.299) The calculated increase in AgtR1a was 100.3% in comparison to control. AgtR2 (angiotensin II type 2 receptor; T_28_=0.5307), TRPV1 (transient receptor potential vanilloid 1 channel; T_28_=0.2589), ACE (angiotensin-converting enzyme; T_16_=0.4231), angiotensinogen (T_16_=1.056), renin (T_16_=0.1950), and regulatory genes of EPAS1 (endothelial PAS domain-containing protein 1)/Hif2α (hypoxia-inducible factor 2 alpha; T_28_=0.3774) and tyrosine hydroxylase (TH-common PC12 and glomus cell marker, T_28_=0.6274) were unchanged. Data presented as difference in cycle threshold (ΔCt) compared with the HPRT (hypoxanthine-guanine phosphoribosyltransferase) housekeeping gene. n=15 per group. Each n represents 1 well. **B**, AgtR1 DNA was hypermethylated in PC12 cells following 6 hours of nicotine exposure. T_10_=3.881. n=5, control; n=6 nicotine. Each n represents 1 well. **C**, PC12 cells were cultured for 24 hours, and cells were prepared for immunoblot. AgtR1 and actin were stained on the same membrane. Quantification of AgtR1 was relative to actin; n=5, control; n=4, nicotine. Two-sided *t* test; T_7_=3.050. **D,** PC12 cells were cultured for 1 hour with media (control, n=5), nicotine (Nic, 50 µg/mL; n=6), angiotensin (Ang, 50 ng/mL; n=5), Nic+Ang (n=7), Ang+Losartan (AII1R blockade, 3 µM; n=8), Nic+Ang+Losartan (n=8), Ang+TRPV1 antagonist (TRPV1x, AMG9810, 10 µM; n=5), Nic+Ang+TRPV1x (n=7), Ang+Losartan+TRPV1x (n=5), and Nic+Ang+Losartan+TRPV1x (n=8) and imaged for calcium flux using Incucyte calcium imaging with Calcium Orange (nonratiometric imaging). 1-way ANOVA (F_9,54_=5.225; *P*<0.0001) Holm-Sidak post hoc test; *P* values are included in the figure.

To show that nicotine itself is responsible for AgtR1 upregulation, we used a cell culture model. PC12 cells have been routinely used as a surrogate CB glomus cell system.^45–47^ Six hours of nicotine exposure significantly upregulated AgtR1a gene expression (Figure 3A). Differences in AgtR2 and TRPV1 were not demonstrated (Figure 3A). Differences in angiotensinogen, angiotensin-converting enzyme, renin, and regulatory genes of EPAS1 (Hif2α) and TH (Figure 3A) were not demonstrated between conditions in relation to the housekeeping gene HPRT. DNA methylation of AgtR1 was confirmed with the DNA methylation pulldown assay (Figure 3B). We confirmed that nicotine exposure increases AgtR1 protein expression with standard immunoblot methods (Figure 3C; Figure S4).

Using calcium imaging, we investigated how nicotine would augment glomus cell excitation. We show that with 1 hour of exposure to nicotine, PC12 cells increased their level of calcium spike frequency. The addition of angiotensin increased PC12 cell firing frequency to a level similar to nicotine. Importantly, nicotine combined with angiotensin did not further increase PC12 cell excitation. Losartan reduced calcium activity in PC12 cells when delivered to angiotensin or nicotine+angiotensin-stimulated cells concurrently. The reduction in calcium flux was also reduced with AMG9810 and in combination with AMG9810 and Losartan (Figure 3D). These results are congruent with our previous data^48^ and others,^49^ showing that TRPV1 is phosphorylated by various protein kinase C isoforms (PKCs) from upstream G-protein coupled receptors. This suggests that TRPV1 phosphorylation by PKCs stimulated by AgtR1 would increase PC12/glomus cell excitation to carry out further downstream efferent nerve excitation. Nicotine has a stimulatory effect on PC12 and, quite likely, CB glomus cells to increase the angiotensin signaling pathway via AgtR1 and TRPV1.

### Angiotensin Acutely Increases Arterial Pressure

**Figure 4.**
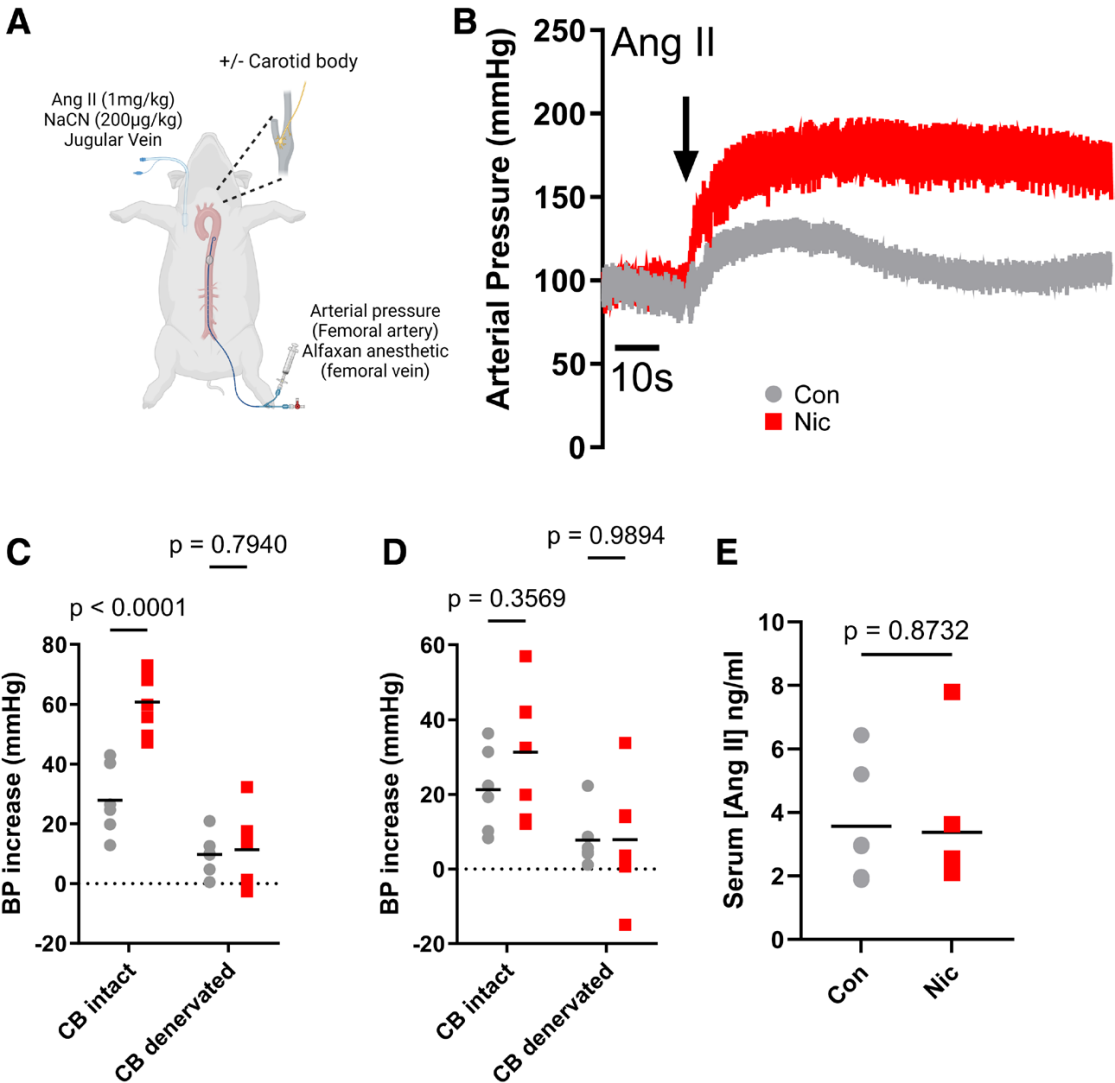
**Carotid body (CB) reactivity to Ang (angiotensin) II is increased in perinatal nicotine (Nic)-exposed pups compared with control (Con) with unaltered serum Ang. A**, Diagram of in vivo experimental preparation; serum was isolated before injection of Ang or sodium cyanide (NaCN). n=6, pre/post. **B**, A representative trace of arterial pressure of vehicle and perinatal Nic-exposed pups on postnatal day (PND) 56 in response to a bolus jugular vein IV injection of Ang. **C**, The absolute increase in the arterial pressure in response to Ang in the vehicle (gray) and perinatal Nic-exposed pups (red) before and following CB denervation (F_1,__11_=11.34). **D**, The absolute increase in arterial pressure in response to NaCN in the vehicle (gray) and perinatal Nic-exposed pups (red) before and following CB denervation (F_1,__11_=2.364). **E**, Serum was harvested before administration of bolus injections (T_10_=0.1637).

To assess acute CB reactivity to angiotensin, pups from perinatal nicotine-exposed or control mothers were anesthetized and given a bolus angiotensin injection into the jugular vein before and after acute CB denervation (Figure 4A and 4B). In response to the jugular vein injected angiotensin (circulates through the pulmonary circulation and then encounters the carotid bodies), arterial pressure was significantly elevated in nicotine-exposed offspring compared with controls (Figure 4C; Figure S5). In response to sodium cyanide, which blocks mitochondrial complex III and has been routinely used as a CB stimulant, there was no significant difference between groups (Figure 4D; Figure S5). Following CB denervation, arterial pressure was suppressed and did not respond to angiotensin or sodium cyanide as vigorously (Figure 4C and 4D; Figure S5). The reactivity to angiotensin and sodium cyanide was attributed to the carotid bodies; however, given that boluses were delivered through the jugular vein, off-target effects cannot be ruled out.

Importantly, serum harvested from these rats before any drug delivery demonstrated that circulating levels of angiotensin were not altered between groups (Figure 4E), similar to previous results.^18^ It is possible that our analysis may be affected by background proteins in our sample, despite the ability of Ang II ELISAs to discern various forms of angiotensin and cleaved fragments.^50^

### Carotid Bodies Are Responsible for Elevated Blood Pressure in Perinatal Nicotine-Exposed Offspring

**Figure 5.**
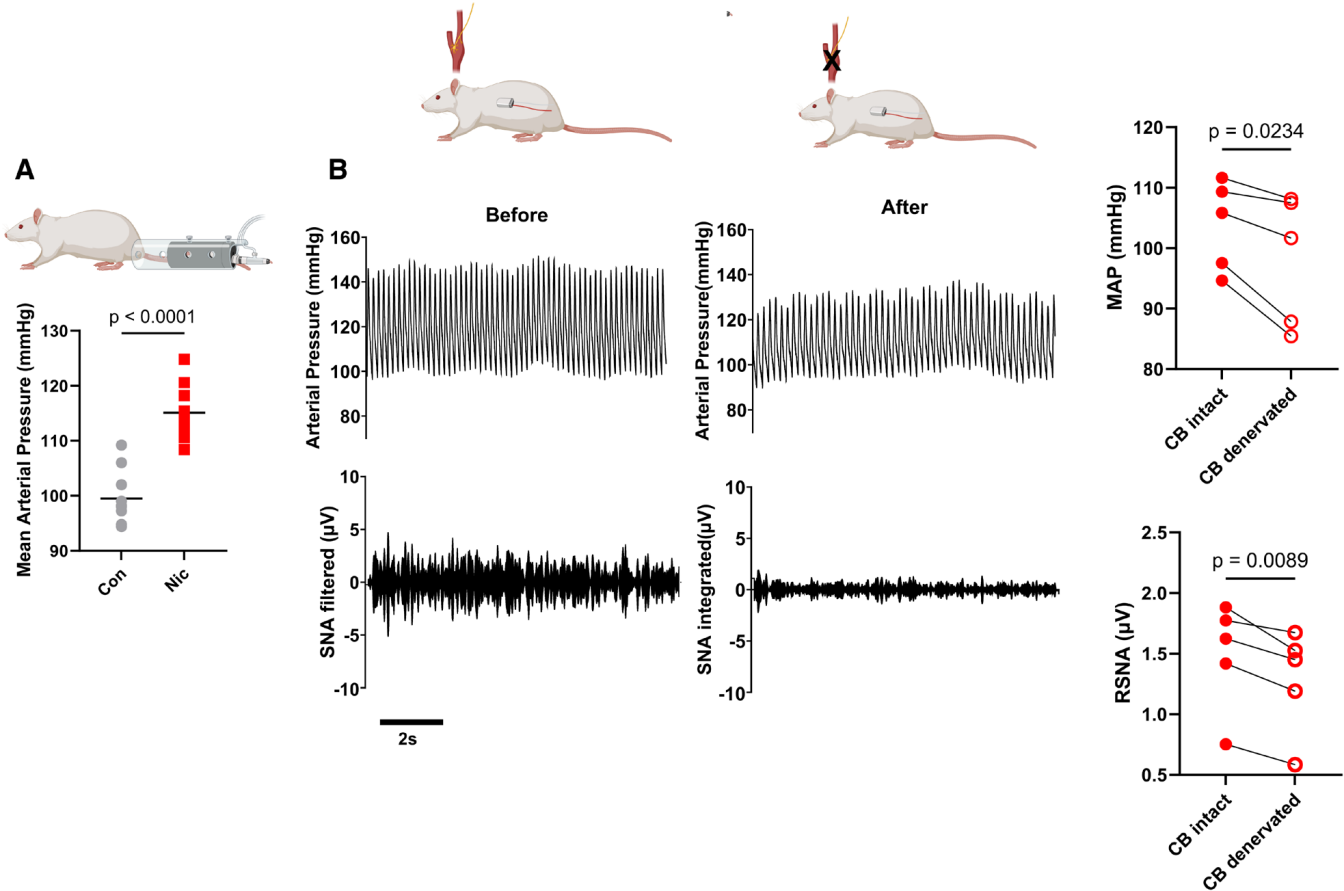
**Perinatal nicotine-exposed rats have increased mean arterial pressure (MAP) mediated by carotid body (CB) augmentation of sympathetic activity. A**, MAP, as assessed by tail-cuff plethysmography, was augmented in nicotine-exposed offspring compared with controls (T_20_=7.964). n=11 per group, 2 independent experiments. **B**, Perinatal nicotine-exposed pups were implanted with radio telemetry to measure arterial pressure (AP) and renal sympathetic nerve activity (RSNA) on PND 56 as per Figure 1A. AP and RSNA were recorded for 7 days, and then, carotid bodies were denervated and recordings recommenced for an additional 7 days. Raw traces of AP and efferent RSNA (vasoconstrictor outflow to kidneys) before CB denervation (CB intact-BEFORE) and following CB denervation (CB denervated-AFTER) in a representative perinatal nicotine exposed rat using telemetry. Summary statistics in n=5 perinatal nicotine rats comparing the absolute change of MAP and RSNA. MAP (T_4_=3.568) and RSNA (T_4_=4.762).

We evaluated arterial pressure between quiescent conscious control and nicotine-exposed offspring using tail-cuff plethysmography on PND 56. Indeed, arterial pressure was higher in nicotine-exposed offspring (Figure 5A). To demonstrate that the carotid bodies were involved in perinatal nicotine-exposed hypertension, we implanted indwelling telemetry to measure real-time arterial pressure from the aorta and efferent sympathetic activity directed to the renal nerve between PNDs 56 and 60 (Figure 5B; Figure S6). We show that within the same animal, CB denervation reduces arterial pressure and RSNA (Figure 5B; Figure S6). The reduction in arterial pressure with CB denervation was predominantly due to the suppression of systolic pressure, which significantly reduced pulse pressure (Figure S6). The reductions of RSNA, likely as a result of CB denervation, also coincide with a reduced respiratory rate, similar to other models of hypertension.^19^ We attribute our changes in response to CB denervation to a reduction of RSNA as measured and demonstrated by others.^19,20^ However, we did not assess the precise contribution of the carotid bodies to RSNA levels as previously.^19^ Nevertheless, we note the association of RSNA to respiratory rate, which indicates that the reduction of respiratory rate following CB denervation would be tied to the fall in RSNA.

## Discussion

In response to adverse maternal programming, epigenetic changes lead to cardiovascular sequelae in later life. Hypertension has been a consistent outcome associated with many adverse intrauterine insults ranging from undernutrition,^51,52^ overnutrition,^52,53^ hypoxia,^54,55^ and stress.^56^ The risk of hypertension is thought to be significantly increased in perinatal nicotine-exposed offspring. The current data demonstrate that epigenetic changes in angiotensin signaling in the carotid bodies contribute to augmented blood pressure in perinatal nicotine-exposed offspring.

We show that epigenetic modifications in response to adverse maternal programming in the form of nicotine exposure increase angiotensin, AgtR1a transcripts, and AgtR1 DNA intron methylation, demonstrating an increase in angiotensin signaling in the carotid bodies. These epigenetic changes in vivo are confirmed in vitro as we show that nicotine directly increases AgtR1 gene expression, AgtR1 DNA methylation, AgtR1 protein, and AgtR1 stimulation in PC12, glomus-like cells, in culture.

Importantly, the difference in epigenetic changes to AgtR1 was predominantly confined to carotid bodies as the brain did not show an increase in gene expression or DNA methylation, whereas kidneys only showed increased DNA methylation between perinatal nicotine exposed and control pups. Angiotensinogen and angiotensin-converting enzyme gene expression was upregulated in the brains and kidneys, and renin was increased in the kidneys of perinatal nicotine exposed compared with control pups. In line with previous investigations, blood pressure reactivity to angiotensin is increased through a neurogenic mechanism involving CB-mediated excitation of efferent sympathetic activity. Finally, we showed, for the first time, that CB denervation reduced blood pressure in perinatal nicotine-exposed rats alongside efferent sympathetic activity.

Clinical data demonstrate that hypertension is likely in offspring that experienced intrauterine/perinatal smoke exposure^2,7,57–61^; however, the age, sex, and potential for additional confounding lifestyle factors make it difficult to tease out this association, and measurements beyond adolescence have not been completed to date. Despite the importance of the perinatal environment on cardiovascular health, knowledge of the patients’ perinatal history appears to be inconsistently applied to clinical decisions.

Preclinical data using rodent models show that vascular reactivity to angiotensin via AgtR1 gene expression, protein expression, and signaling increases in aortae and mesenteric arterioles following prenatal and perinatal nicotine exposure.^14,15,18^ Accordingly, increased AgtR1 activity, RNA, and DNA methylation^62,63^ have been consistent findings in response to perinatal nicotine-exposed offspring in vascular tissues. Our data show that additional sensory neural systems, which are significantly tied to hypertension,^20^ are also augmented and provide another mechanism for hypertension in perinatal nicotine-exposed rats.

To date, scant data are available regarding the effects of nicotine exposure or perinatal nicotine exposure on CB activity and resulting autonomic reflex disturbances.^23,64^ Carotid bodies are tasked with a multitude of homeostatic control mechanisms that regulate nutrient and oxygen delivery to tissues.^21^ Their role in blood pressure regulation is highlighted by genetic^22^ and epigenetic^27^ modifications demonstrating the ability to increase basal activity and result in augmented blood pressure. Specifically, the spontaneously hypertensive rat has augmented CB basal activity, which translates to alterations in breathing, blood pressure regulation, and glucose homeostasis.^22^ Importantly, these CB changes appear to hinge on a significant difference in transcriptomic profile compared with their normotensive genetic counterpart, the Wistar-Kyoto rat.^22^ Furthermore, and particularly salient to the current argument, CB denervation in spontaneously hypertensive rats reduced arterial pressure and respiratory modulation, whereas CB denervation in Wistar-Kyoto rats had no effect.^19^ Importantly, evidence demonstrates that CB basal excitation can change via epigenetic modifications that suppress redox disinhibition^27^ to alter basal regulatory mechanisms such as breathing regulation.

A consistent finding is that intrauterine conditions with low oxygen^23^ or hyperleptinemia^65^ lead to augmented CB basal activity. Therefore, intrauterine environmental deficiencies may augment offspring arterial pressure due to a CB-regulated mechanism. Our data are among the first to demonstrate that adverse maternal programming may contribute to CB epigenetic changes in AgtR1a, and importantly, these epigenetic changes occur as a result of genetic upregulation via DNA methylation, which likely leads to CB excitation. Our in vitro experiments with PC12 cells demonstrate epigenetic changes in CB angiotensin signaling of nicotine exposure, which also increases protein expression of AgtR1. Furthermore, the reactivity of PC12 cells was regulated by nicotine to increase AgtR1 signaling. AgtR1 signaling in the carotid bodies has been tied to a TRPV1-mediated mechanism in response to repeated hypoxic bouts.^44^ Given that losartan and TRPV1 blockade suppressed calcium signaling in PC12 cells, with no further reduction in calcium excitation with dual inhibition, we suggest that AgtR1 stimulation of PKC leads to TRPV1 phosphorylation, which increases the propensity for cellular depolarization providing the stimulus for CB excitation and downstream efferent sympathetic signaling. This suggestion is consistent with previous data showing that TRPV1 is responsible, in part, for regulating blood pressure in a 2-kidney, 1-clip model of hypertension^66^ through an alternate neural mechanism. These data fit with the increased CB reactivity to angiotensin in an anesthetized preparation and the consistent reduction in arterial pressure with CB denervation in conscious perinatal nicotine-exposed offspring.

### Limitations

This study did not target the AgtR1-TRPV1 pathway directly in the carotid bodies of perinatal nicotine-exposed pups. Transgenic rat models to specifically target AgtR1 in rats are not readily available. Future investigation into tissue-specific suppression techniques in vivo to confirm the findings in this article is warranted. Furthermore, we note that certain antibodies for the AgtR1 receptor have shown a lack of specificity in mice.^67^ Whether this finding is extended to rats has not been demonstrated to the authors’ knowledge. However, the upregulation of the AgtR1-TRPV1 pathway in the carotid bodies of perinatal nicotine-exposed offspring was probed with multiple orthogonal methods in vitro and in vivo.

### Conclusions

Epigenetic modifications within the carotid bodies contribute, in part, to elevated arterial pressure resulting from perinatal nicotine exposure. The increased CB AgtR1-TRPV1 signaling pathway likely contributes to efferent sympathetic activity to augment arterial pressure.

### Perspectives

CB denervation has shown clinical utility, as human clinical trials have shown that unilateral CB denervation results in a meaningful reduction in human blood pressure.^68,69^ In fact, the reduction of systolic and diastolic arterial pressures with CB unilateral denervation^69,70^ appears to be similar to renal denervation.^71,72^ Although effective, alternate methods to target neurogenic hypertension should be encouraged, given the risk associated with such surgical methods.

Targeting the carotid bodies offers the advantage of suppressing the first step of reflex blood pressure regulation (ie, sensory afferents^71,72^). We suggest that a targeted suppression or reversal of angiotensin signaling in the carotid bodies and renin-angiotensinogen and angiotensin-converting enzymes in the kidneys would help alleviate increased blood pressure and cardiac afterload and limit unwanted side effects by standard drug therapies or invasive procedures. Given the advent of new delivery strategies that target specific tissues,^73^ the unwanted side effects of current medications could be reduced as therapies would be delivered where needed, in smaller amounts.

## Article Information

### Acknowledgments

The content is solely the responsibility of the authors and does not necessarily represent the official views of the National Institutes of Health or the University of California Office of the President Tobacco-Related Diseases Research Program. Figures 1A, 2A, 4A, 6A, and 6B and the graphical abstract were completed with Biorender under a CC-BY-NC-ND license.

### Author Contributions

F. Zhu, Z. Wang, and J. Zyuzin contributed to the experimental design, conducted experiments, and analyzed data. H. McSwiggin, K. Davis, and J. Liu contributed to the experimental execution and data analysis. W. Yan and V.K. Rehan assisted with experimental design and analysis. N. Jendzjowsky designed the study, conducted experiments, analyzed data, and prepared the article and figures. All authors agree with the article.

### Sources of Funding

This project was supported by the grant T32IP4707 of the Regents of the University of California Tobacco-Related Diseases Research Program (TRDRP, to N. Jendzjowsky). W. Yan is supported by National Institutes of Health (NIH) grants P50HD098593 and R01HD099924. V.K. Rehan is supported by NIH grants R01HL151769 and IR00737, T32IP5044, T32IR5048, and T32IR5365 of the Regents of the University of California TRDRP. Z. Wang was supported by a California Institute for Regenerative Medicine Stem Cell Biology Training Grant EDUC4-12837.

### Disclosures

None.

### Supplemental Material

Expanded Methods

Figures S1–S6

Data S1–S3

References 28–42

## Supplementary Material
