# Supplementary material for: Epigenetic Upregulation of Carotid Body Angiotensin Signaling Increases Blood Pressure
Source: Hypertension. 2024 Dec 5;82(2):293–305. doi: 10.1161/HYPERTENSIONAHA.124.23349 (PMC11732265; doi:10.1161/HYPERTENSIONAHA.124.23349)
Supplement: Supplementary file 1 [file hyp-82-293-s001.pdf]

Supplementary materials

Epigenetic upregulation of carotid body angiotensin signaling increases blood pressure.

Fengli Zhu M.Sc.[1], Zhuqing Wang Ph.D.[1], Kayla Davis B.Sc.[1], Hayden McSwiggin B.Sc.[1], Jekaterina Zyuzin Ph.D.[1], Jie Liu Ph.D.[1], Wei Yan M.D. Ph.D.[1, 2, 5], Virender K. Rehan M.D.[1, 3, 5], Nicholas Jendzjowsky Ph.D.[1, 4, 5*]

[1]The Lundquist Institute for Biomedical Innovation at Harbor-UCLA Medical Center, Torrance, CA 90502, USA
[2]Division of Metabolic Diseases and Translational Genomics, Harbor-UCLA Medical Center, Torrance, CA 90502
[3]Division of Neonatology, Harbor-UCLA Medical Center, Torrance, CA 90502, USA
[4] Division of Respiratory and Critical Care Medicine and Physiology, Harbor-UCLA Medical Center, Torrance, CA 90502, USA
[5]David Geffen School of Medicine, UCLA, Los Angeles, CA 90073, USA

Short title: Epigenetic programming of carotid bodies

*Corresponding Author: Nicholas Jendzjowsky, PhD
Rm 209 Martin Research Building,
The Lundquist Institute for Biomedical Innovation at Harbor-UCLA Medical Center
Division of Respiratory and Critical Care Medicine and Physiology
David Geffen School of Medicine, UCLA
1124 W Carson Street
Torrance, CA
90502-2006
Office:323-457-1663
Email: nicholas.jendzjowsky@lundquist.org

DETAILED MATERIALS AND METHODS

Data availability

RNA sequencing and DNA bisulfite sequencing data have been deposited and can be found in the NIH SRA (Bioproject ID: PRJNA1106547). All other data are found in the supporting data and supplementary files and can be further made available upon request to the corresponding author.

Animal models, care, and ethical standards

First-time pregnant Sprague-Dawley rat dams were purchased from Charles River (Hollister, CA) at gestational day 3. SD mothers were subject to subcutaneous nicotine injections (2mg/kg, Millipore Sigma, PHR2532) from gestational day 7 to post-natal day (PND) 21[1–3]. Control mothers received PBS (vehicle). Animals were housed with controlled temperature (21 ± 2 °C), humidity (55 ± 10%) and 12h light-dark cycle. Pups were weaned on post-natal day (PND) 21 and housed in pairs. Animals had unlimited access to food and water. Experiments on pups took place from PND56-80. All experimental procedures were approved by The Lundquist Institute for Biomedical Innovation at Harbor UCLA Medical Center IACUC board, protocol # 31659.

Assignment of control or nicotine to mothers was block randomized to minimize experimenter bias. Unbiased transcriptomic and genomic sequencing was based on previous data acquired by neuronal sequencing results. *In vitro* and *in vivo* power calculations were based on an estimated effect size of 50% difference with assumed unified standard deviation across groups.

PC12 culture

PC12 cells were cultured in RPMI 1640 (Gibco # 11875093) supplemented with 10% Horse Serum (Gibco Cat. #26050-088) and 5% fetal bovine serum (Gibco Cat. #F2442), 0.4µg/ml dexamethasone (Millipore Sigma Cat. # D4902) and 1% penicillin/streptomycin (Millipore Sigma Cat. # P0781). Cells were seeded at $3x10^5$ cells in 1ml of media per well (24 well plate, for qPCR), $2x10^6$ cells in 2ml of media per well (6 well plate, for immunoblot) or 2500 cells in 150µl of media per well (96 well plate, for calcium imaging). Cells were incubated for 6 hours (qPCR and DNA methylation) or 24 hours (immunoblot) with or without nicotine (50µg/ml).

Next-generation RNA sequencing

On PND 56, animals were sedated (5% isoflurane, balance O2), exsanguinated, and carotid bodies gross dissected and placed in a sylgard-coated dissecting dish filled with Ham's F12 media. Carotid bodies were then freed from the carotid bifurcation under a dissecting microscope. Five carotid body pairs (n=10 carotid bodies from 5 rats) from each group and sex (n=5 rats per group, per sex) were lysed, and RNA was isolated using the easy-spin Total RNA extraction kit (Boca scientific, Cat. # 17221). RNA purity was tested with Nanodrop spectrophotometer (Thermofisher Cat. # ND2000USCAN) and deemed appropriate if A260/280 was between 1.99 and 2.10. RNA sequencing was then conducted by an unbiased commercial vendor (Novogene corporation, Sacramento, CA), where further RNA purity was analyzed (RNA integrity number >7). RNA sequencing was performed by Novogene Corporation Inc. (Sacramento, USA). mRNA was purified from total RNA using poly-T oligo-attached magnetic beads. The first cDNA strand was synthesized using a random hexamer primer and M-MuLV Reverse Transcriptase (RNase H−) to generate the cDNA library. Second-strand cDNA synthesis was subsequently performed using DNA Polymerase I and RNase H. Double-stranded cDNA was purified using AMPure XP beads, and the remaining overhangs of the purified double-

stranded cDNA were converted into blunt ends via exonuclease/polymerase. After 3' end adenylation, a NEBNext Adaptor with a hairpin loop structure was ligated to prepare for hybridization. To select cDNA fragments of 150–200 bp in length, the library fragments were purified with the AMPure XP system (Beckman Coulter, Beverly, USA). Finally, PCR amplification was performed, and PCR products were purified using AMPure XP beads. The samples were sequenced on an Illumina NovaSeq 6000 with ≥20 million read pairs per sample. The Novogene Corporation conducted full RNA-seq analysis with their pipeline which included alignment with HISAT2(49), PCA on the gene expression values (FPKM), differential gene expression (DEG) analysis using DESeq2(50), and a functional analysis including Gene ontology (GO) and Kyoto Encyclopedia of Genes and Genomes ontology (KEGG).

Whole genome DNA-bisulfite sequencing

Carotid bodies were dissected as above. Five carotid body pairs (n=10 carotid bodies from 5 rats) from each group and sex (n=5 rats per group, per sex) were lysed and DNA was isolated using the QIAamp UCP DNA Micro Kit (Qiagen, Cat. # 56204). DNA purity was deemed sufficient, with A260/280 between 1.75-1.85. Bisulfite conversion and downstream whole-genome DNA-bisulfite sequencing were conducted by (Novogene Corporation, Sacramento, CA).

The reference genome was transformed into a bisulfite-converted version (C-to-T and G-to-A converted) and then indexed using bowtie2[4]. Sequence reads were transformed into fully bisulfite-converted versions (C-to-T and G-to-A converted) before alignment to the converted versions of the genome in a directional manner. Sequence reads were compared to the normal genomic sequence, and all cytosine position methylation states were inferred. Methylation results were transformed into bigWig format for visualization using IGV browser. The sodium bisulfite non-conversion rate was calculated as the percentage of cytosine sequenced at cytosine reference positions in the lambda genome. The sum of methylated and unmethylated read counts was calculated as methylated reads divided by the methylated plus total reads. Differentially methylated regions (DMR) were identified using DSS software[5–7]. The GOseq R package implemented enrichment analysis of genes related to DMRs[8], in which gene length bias was corrected. GO terms with corrected p-values less than 0.05 were considered significantly enriched by DMR-related genes. KEGG[9] pathways statistical enrichment of DMR-related genes were analyzed with KOBAS software[10].

RNA qPCR of carotid bodies

For carotid bodies, an additional group of n=3 samples (n=5 rats per sample) from each group was harvested as above, snap frozen, and stored until analysis. Brain and kidneys were harvested from each group of pups (one tissue per pup was deemed as n=1). PC12 cells were washed in PBS, then immediately lysed in lysis buffer, and converted into cDNA as above. N=3 wells were analyzed for each condition for each probe and five separate experiments were run. RNA was converted to cDNA using the Tetro cDNA synthesis kit (Meridian life sciences, Cat. # Bio65043). qPCR was conducted with Taqman specific probes. AgtR1a (Thermofisher Rn02758772_s1), AgtR2 (Thermofisher Rn00560677_s1), angiotensinogen (Thermofisher RN00593114_m1), angiotensin-converting enzyme (Thermofisher Rn00561094_m1), renin (Thermofisher Rn02586313_m1), TH (Thermofisher Rn00562500_m1), endothelial PAS domain-containing protein 1/ hypoxia inducible factor 2 alpha (EPAS1/Hif2α, Rn00576515_m1) and TRPV1 (Thermofisher Rn00583117_m1). All experimental genes were evaluated in reference to hypoxanthine-guanine phosphoribosyltransferase (HPRT Thermofisher

Rn01527840_m1). The qPCR reaction was completed using Taqman fast advanced master mix enzyme (Thermofisher Cat. # 4444557) and the QuantStudio 3 thermocycler (Thermofisher Cat. #A28567). Delta CT was calculated in reference to HPRT, and comparisons between groups were made using an unpaired two-sided t-test.

Methylated DNA immunoprecipitation-QPCR (MeDIP-qPCR)

DNA was isolated from carotid bodies (n=4 per group, each n is comprised of carotid bodies from 5 rats), brains, and kidneys (n=4 rats per group, one tissue is n=1), and PC12 cells (1 well is n=1). Carotid body and PC12 DNA were isolated using the QIAamp UCP DNA Micro Kit (Qiagen, Cat. # 56204). Brain and kidney DNA was isolated using the Puregene Tissue kit (Qiagen, Cat. # 158063). MeDIP was performed using the Methylated DNA immunoprecipitation kit (Cat.# ab117133, Abcam) following the procedures described previously[11]. Briefly, 100µL of the antibody buffer and 1µL anti-5-methylcytosine or reference IgG antibody were added into wells and incubated at room temperature for 1 hour. During the incubation, 1µg of genomic DNA was sheared between 200 and 1000 bp in the reaction buffer using the Covaris M220 ultrasonicator, followed by denaturation at 95 °C for 2 minutes, then placed on ice until use. An aliquot of 5 µL of the denatured DNA was saved as the input DNA. The strip wells bound with antibodies were washed with 150µL of the antibody buffer and 150µL of the wash buffer once, followed by incubation with the sheared DNA at room temperature for 2 hours. The antibody-enriched DNA was eluted in the DNA release buffer containing proteinase K followed by DNA purification. qPCR was performed using Fast SYBR™ Green Master Mix (Cat.# 4385612, Thermo Fisher Scientific) to identify DNA methylation levels. Primer sequence-AgtR1: Forward TACCTAAACATAGTAAAGCCAAACACA; AgtR1: Reverse TATCCTGTTGATCTCTTTTTGTTGTCTG. ΔCt was calculated as Methylated AgtR1 DNA – Total AgtR1 DNA and was used to plot the data[11,12]. Comparisons between groups were made using an unpaired two-sided t-test.

Immunoblot of PC12 cells

After incubation with or without nicotine (above), PC12 cells were lysed with RIPA buffer (500µl/well, Thermoscientific Cat. #89901) containing protease and phosphatase inhibitors (5µl/well, Thermoscientific Cat. #78442) and stored at -80°C until analysis. Five µl of sample for each replicate was loaded onto 4-12% Bis-Tris gels (Invitrogen Cat. #NW04120BOX) with ladder (Thermoscientific Cat. #26616) and separated for 45 minutes at 170 volts. Gels were transferred onto PVDF membrane (MilliporeSigma Cat. #IPVH00010) for 1 hour at 20 volts. Membranes were blocked for 1 hour at room temperature with 5% non-fat milk powder (Lab Scientific bioKEMIX Cat. #M0841) in TBST (Fisherbioreagents Cat. #BP2471-1). Membranes were incubated with actin primary antibody (Sigma Cat. #A5441-2ml, 1:5000) for 1 hour and then incubated with secondary goat-anti mouse antibody (Invitrogen Cat. #31430, 1:5000) for 1 hour. Blots were incubated with Super signal Chemiluminescent substrate (Thermoscientific Cat. #34577) for 5 minutes and imaged Biorad Gel imager (Chemidoc MP imaging system). Blots were stripped with HRP stripping buffer (Azure biosystems Cat. #AC2154) and re-incubated with AgtR1 primary antibody (Proteintech Cat. #25343-1-AP, 1:600) for 1 hour at room temperature and then incubated with secondary goat anti-rabbit antibody (Invitrogen Cat. #31460, 1:5000) for 1 hour at room temperature. Blots were then incubated with Super signal Chemiluminescent substrate (Thermoscientific Cat. #34577) for 5 minutes and again imaged with the Biorad Gel imager (Chemidoc MP imaging system). Bands were quantified with Biorad

ImageLab and the AgtR1 signal was normalized to actin. Groups were compared using Student's two-sided t-test.

Immunohistochemistry

Carotid bodies were gross dissected as above. Then they were cleaned from connective tissue but left attached to the surrounding carotid artery bifurcation as previously[13]. Tissues were fixed in 4% paraformaldehyde for 1-2 hours at 4°C, then cryopreserved in 30% sucrose overnight at 4°C, dried embedded in optimal cutting temperature compound, and cut at 14μm sections with a cryostat. Sections were then washed (PBS), permeabilized (Triton x 20%), blocked with 10% goat serum (Abcam, Cat. # ab7481), and incubated with antibodies for tyrosine hydroxylase (TH, Millipore Sigma ZMS1033, clone 20/40/15), AgtR1 (Millipore Sigma AB15552, polyclonal) and TRPV1 (Alomone Labs ACC-030, polyconal) for 24hours at 4°C and subsequently stained with appropriate secondaries (Jackson Laboratories Goat-anti rabbit Cy3 111-165-003, Cy5 111-005-003 and Goat-anti mouse 488 115-545-003) for 90 minutes and then counterstained with DAPI (Millipore Sigma MBD0015) and coverslipped with prolong diamond antifade mounting media (Thermofisher #P36970). Slides were imaged using the Leica Thunder imaging acquisition system.

Calcium imaging

PC12 cells were separated into media (control), stimulated with nicotine (Sigma Cat. # 6019-06-3, 50μg/ml), angiotensin II (Tocris Cat. # 1158, 5μM), nicotine + angiotensin II, angiotensin II + losartan (Tocris Cat. # 3798, 3μM), nicotine + angiotensin II + losartan, angiotensin II + AMG9810 (TRPV1 antagonist Tocris Cat. # 2316, 10μM), nicotine + angiotensin II + AMG9810, or nicotine + angiotensin II + losartan + AMG9810, for 1 hour and then incubated with Calcium Orange AM methyl ester (Invitrogen Cat. # C3015) + 0.01% Pluronic acid (Invitrogen Cat. #F-127) for 20 minutes at 37°C 5%CO_2, washed in media and then imaged in the Incucyte imaging system (Sartorius) using the Neural imaging parameters (Ex: 549λm/ Em: 576λm, 60s) for 1 hour. The frequency of methyl ester excitation bursts taken as the frequency of bursts directly from automated Incucyte software and averaged for each well to express a single value. Data were analyzed with One-way ANOVA using the Holm-Sidak post-hoc test.

In vivo carotid body reactivity

Rats from control or nicotine groups (as above) were tested on PND 56-58. Rats were anesthetized with 5% isoflurane (balance room air) using the Kent Scientific Somnosuite anesthesia system. The femoral artery and vein were cannulated (P50 tubing), and the arterial line was instrumented to a pressure transducer to measure continuous arterial pressure (AD instruments MLT0670) using the PowerLab data acquisition console (AD instruments PL3516/P). The vein was then connected to a syringe pump (Fisherscientific Cat. # 14831200) to induce a 15mg/kg/min infusion of alfaxan (Victor Medical Cat. # 1629020)[14,15]. Then the jugular vein was cannulated (P90) to deliver bolus injections of Angiotensin II (Tocris Cat. # 1158 1mg/kg) and sodium cyanide (NaCN, Fisherscientific Cat. # AA1213722, 200μg/kg). These injections were repeated following bilateral carotid body denervation. Briefly, the neck area was shaved, and a midline incision exposed the carotid artery. Then glands were pushed aside, and the carotid bodies were identified within the carotid bifurcation underneath the occipital artery. The carotid sinus nerve was confirmed as it was traced to the glossopharyngeal nerve, then denervated, as previous[14]. Mean arterial pressure was averaged at the 60s preceding each bolus injection and a 60s average during the peak reactive response to each bolus injection. Data were

analyzed by Two-way ANOVA (Group x Carotid body intact vs. denervated) for Angiotensin II or NaCN injection. Holm-Sidak post-hoc test determined group differences.

Serum angiotensin concentration

Blood was collected after induction of IV anesthetic but, before any drug infusion from the above experiment from the femoral vein. Blood was left to coagulate for 20 minutes on ice then spun at 4,000rpm for 10 minutes at 4°C. Serum was drawn, aliquoted, snap-frozen, and stored at -80°C until analysis. Angiotensin ELISA (Raybiotech #EIAR-ANGII-1) was used per manufacturer's instructions. Groups (Control pups vs. perinatal nicotine-exposed pups) were analyzed with two-sided unpaired t-test.

Arterial pressure assessment between groups

Rats from control or perinatal nicotine-exposed mothers were handled daily following weaning at PND 21 and habituated to the tail plethysmography system for 1 hour per day for 2 weeks (IITC Lifesciences). Three-minute averages of tail cuff-measured blood pressure were used to attain a single value for each animal. Data were compared using an unpaired two-sided t-test.

Carotid body-mediated changes in arterial pressure

Perinatal nicotine-exposed rats were prepared for telemetric implant between PND 56-60. Rats were anesthetized with 5% isoflurane (balance O_2) as induction and maintained on a surgical plane with 2-3% isoflurane. The abdominal area was isolated, shaved, and treated with 3x betadyne and 3x 70% alcohol. A midline incision was made, and the descending aorta below the renal arteries and renal nerves were isolated. The Kaha telemetry dual sympathetic nerve activity /pressure (TRM56SP, AD Instruments) was instrumented around the renal nerves using KwikGard dental impression material to isolate electrodes from electrical interference and abdominal movements for renal sympathetic nerve activity (RSNA) recording. The pressure transducer was inserted into the aorta as per manufacturer's instructions utilizing Vetbond to measure continuous pressure measurement. The surgical area was sewn in layers, and the skin was secured with surgical staples. Rats were given buprenorphine (0.03mg/kg SQ) 2x daily for three days and allowed to recover for a total of 5 days before commencing recording. Pressure and RSNA were recorded for one week; then rats were anesthetized as above; the neck area was isolated, shaved, and treated with 3x betadine and 3x 70% alcohol. A midline incision was made on the neck, and the carotid bodies were identified behind the occipital arteries. The carotid sinus nerve was severed bilaterally, and the skin was sewn with subcuticular sutures. Buprenorphine was delivered as above, and recording commenced for another week following recovery. Arterial pressure waveforms delineated heart rate, systolic, diastolic, mean arterial pressure, pulse pressure, and respiratory rate. RSNA was filtered, rectified, and integrated using LabChart 8. Data were binned into daily averages to obtain 7 days of values, pre- and post-carotid body denervation, for each rat and analyzed with a pre-post with a two-sided paired t-test.

Statistics

Specific statistical tests are described for each experiment above. Data were averaged and analyzed for each experiment as described above. Data were analyzed using Graphpad v10.

References

1. Liu J, Yu C, Doherty TM, Akbari O, Allard P, Rehan VK. Perinatal nicotine exposure-induced transgenerational asthma: Effects of reexposure in F1 gestation. *FASEB journal: official*

publication of the Federation of American Societies for Experimental Biology. 2020;34(9):11444–11459.

2. Xiao D, Huang X, Li Y, Dasgupta C, Wang L, Zhang L. Antenatal Antioxidant Prevents Nicotine-Mediated Hypertensive Response in Rat Adult Offspring. *Biology of Reproduction*. 2015;93(3):66.
3. Wongtrakool C, Wang N, Hyde DM, Roman J, Spindel ER. Prenatal Nicotine Exposure Alters Lung Function and Airway Geometry through α7 Nicotinic Receptors. *American Journal of Respiratory Cell and Molecular Biology*. 2012;46(5):695–702.
4. Langmead B, Salzberg SL. Fast gapped-read alignment with Bowtie 2. *Nature Methods*. 2012;9(4):357–359.
5. Feng H, Conneely KN, Wu H. A Bayesian hierarchical model to detect differentially methylated loci from single nucleotide resolution sequencing data. *Nucleic Acids Research*. 2014;42(8):e69.
6. Wu H, Xu T, Feng H, Chen L, Li B, Yao B, Qin Z, Jin P, Conneely KN. Detection of differentially methylated regions from whole-genome bisulfite sequencing data without replicates. *Nucleic Acids Research*. 2015;43(21):e141.
7. Park Y, Wu H. Differential methylation analysis for BS-seq data under general experimental design. *Bioinformatics*. 2016;32(10):1446–1453.
8. Young MD, Wakefield MJ, Smyth GK, Oshlack A. Gene ontology analysis for RNA-seq: accounting for selection bias. *Genome Biology*. 2010;11(2):R14.
9. Kanehisa M, Araki M, Goto S, Hattori M, Hirakawa M, Itoh M, Katayama T, Kawashima S, Okuda S, Tokimatsu T, Yamanishi Y. KEGG for linking genomes to life and the environment. *Nucleic Acids Research*. 2008;36(Database issue):D480-484.
10. Mao X, Cai T, Olyarchuk JG, Wei L. Automated genome annotation and pathway identification using the KEGG Orthology (KO) as a controlled vocabulary. *Bioinformatics (Oxford, England)*. 2005;21(19):3787–3793.
11. Wang Z, McSwiggin H, Newkirk SJ, Wang Y, Oliver D, Tang C, Lee S, Wang S, Yuan S, Zheng H, Ye P, An W, Yan W. Insertion of a chimeric retrotransposon sequence in mouse Axin1 locus causes metastable kinky tail phenotype. *Mobile DNA*. 2019;10(1):17.
12. Lisanti S, von Zglinicki T, Mathers JC. Standardization and quality controls for the methylated DNA immunoprecipitation technique. *Epigenetics*. 2012;7(6):615–625.
13. Jendzjowsky NG, Roy A, Wilson RJA. Asthmatic allergen inhalation sensitises carotid bodies to lysophosphatidic acid. *Journal of Neuroinflammation*. 2021;18:191.
14. Jendzjowsky NG, Roy A, Barioni NO, Kelly MM, Green FHY, Wyatt CN, Pye RL, Tenorio-Lopes L, Wilson RJA. Preventing acute asthmatic symptoms by targeting a neuronal mechanism involving carotid body lysophosphatidic acid receptors. *Nature Communications*. 2018;9(1):4030.
15. Marshall JM. Interactions between local dilator and sympathetic vasoconstrictor influences in skeletal muscle in acute and chronic hypoxia. *Experimental Physiology*. 2015;100(12):1400–1411.

Figure S1.

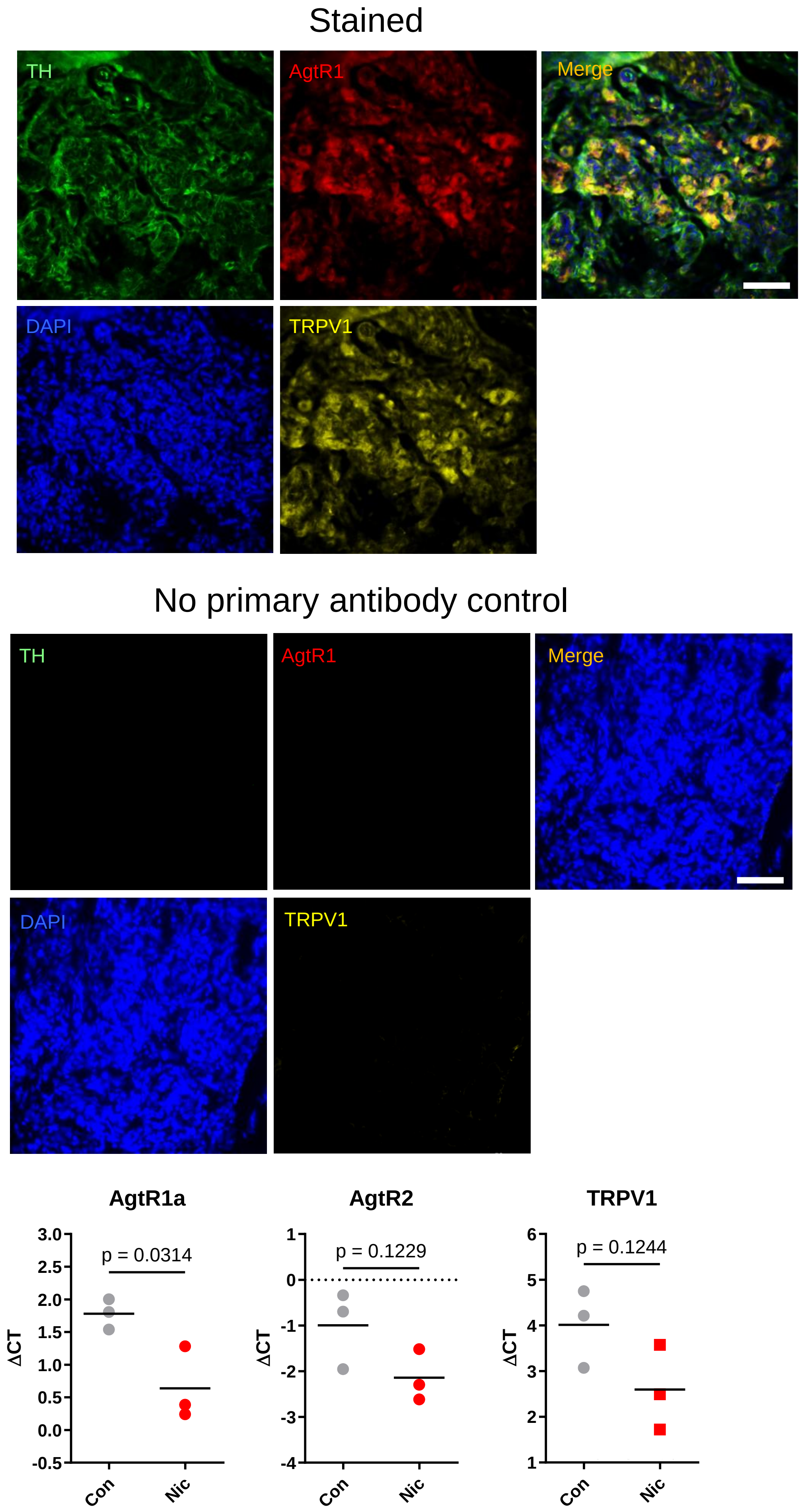

Figure S1. Angiotensin II Type 1 receptor is colocalized with TRPV1 at in the carotid bodies. Angiotensin II type 1 receptor (AgtR1) is colocalized to glomus cells (express tyrosine hydroxylase, TH) and are adjacent to transient receptor potential vanilloid 1 (TRPV1). Scale bar=20µm. Quantitative polymerase chain reaction (qPCR) of carotid bodies (n=3 per sample, n=3 samples per group) shows that AgtR1a is upregulated with perinatal nicotine exposure compared to saline control exposed rats. The calculated increase of AgtR1a was 56.45% in comparison to control. AgtR1a - T_4=2.792; AgtR2 - T_4=1.950; TRPV1-T_4=1.735.

Figure S2.

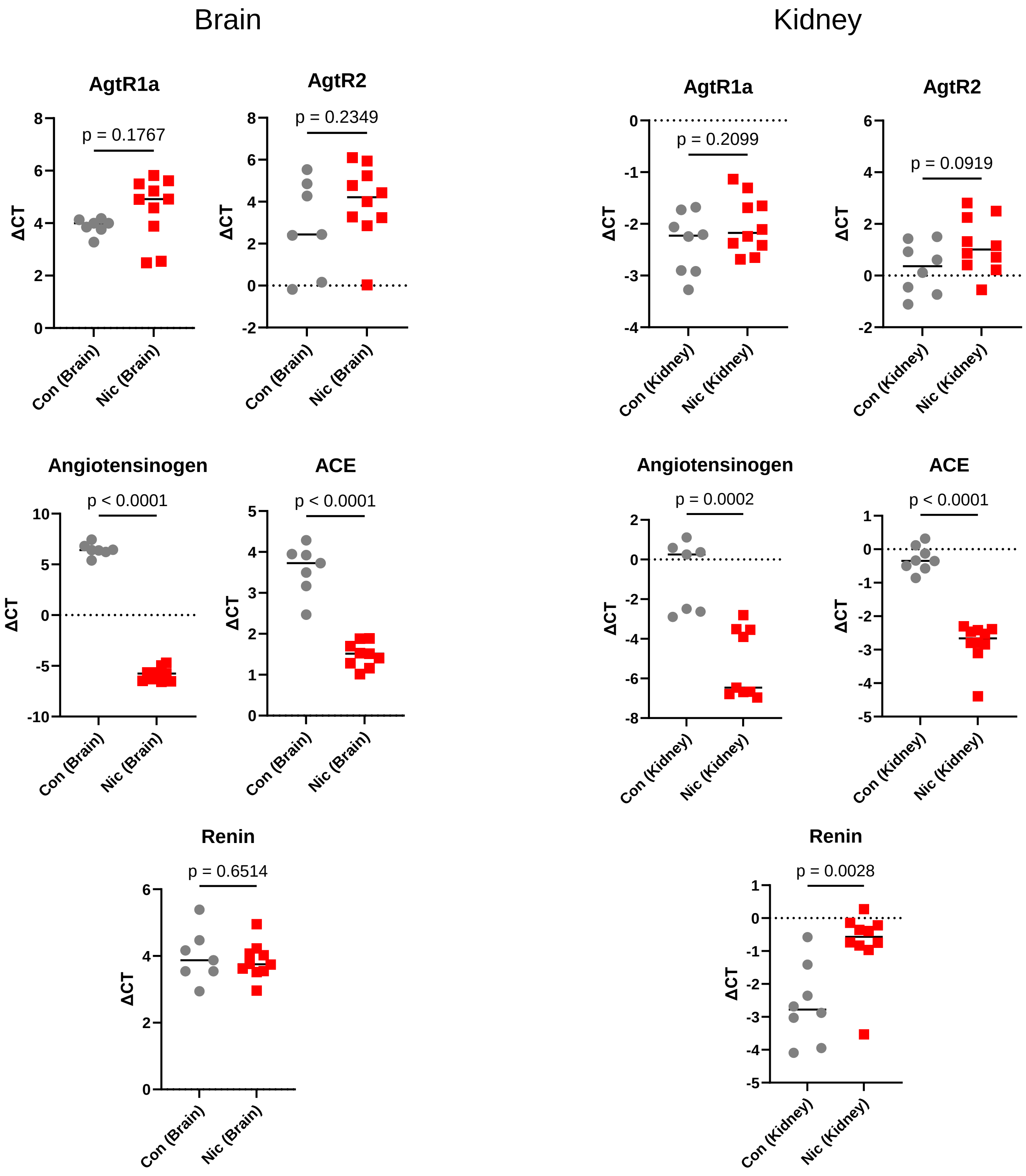

Figure S2. Perinatal nicotine exposure increases gene expression for Angiotensin production in the brain and kidney. Angiotensinogen and angiotensin converting enzyme are upregulated in brains and kidneys of perinatal nicotine exposed rats compared to control rats. Angiotensin receptors are unchanged between groups. Renin, in kidneys, is downregulated in perinatal nicotine exposed rats compared to control. Brain- AgtR1a- T_{15}=1.418, AgtR2- T_{15}=1.238, Angiotensinogen- T_{14}=37.16, angiotensin converting enzyme (ACE)- T_{14}=9.075, Renin- T_{15}=0.4610. Kidney- AgtR1a- T_{16}=1.306, AgtR2- T_{16}=1.793, Angiotensinogen- T_{14}=5.019, angiotensin converting enzyme (ACE)- T_{16}=10.12, Renin- T_{16}=3.529. Brain: control- n=7; nicotine- n=10. Kidney: control- n=8; nicotine- n=10.

Figure S3.

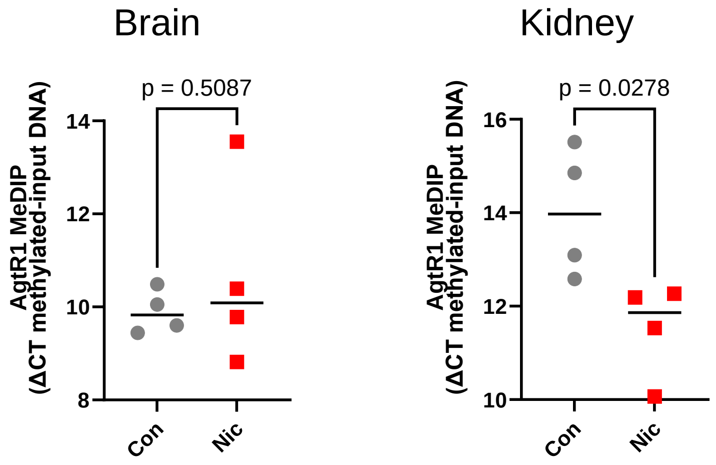

Figure S3. DNA methylation of AgtR1 is confined to carotid bodies. Immunoprecipitation of methylated DNA compared to whole DNA did not reveal differences between perinatal nicotine-exposed and control groups in the brains for AgtR1. AgtR1 methylated DNA was increased in kidneys of perinatal nicotine-exposed rats compared to controls. Brain- T_6=0.0946. Kidney- T_6=1.924. n=4 per group.

Figure S4.

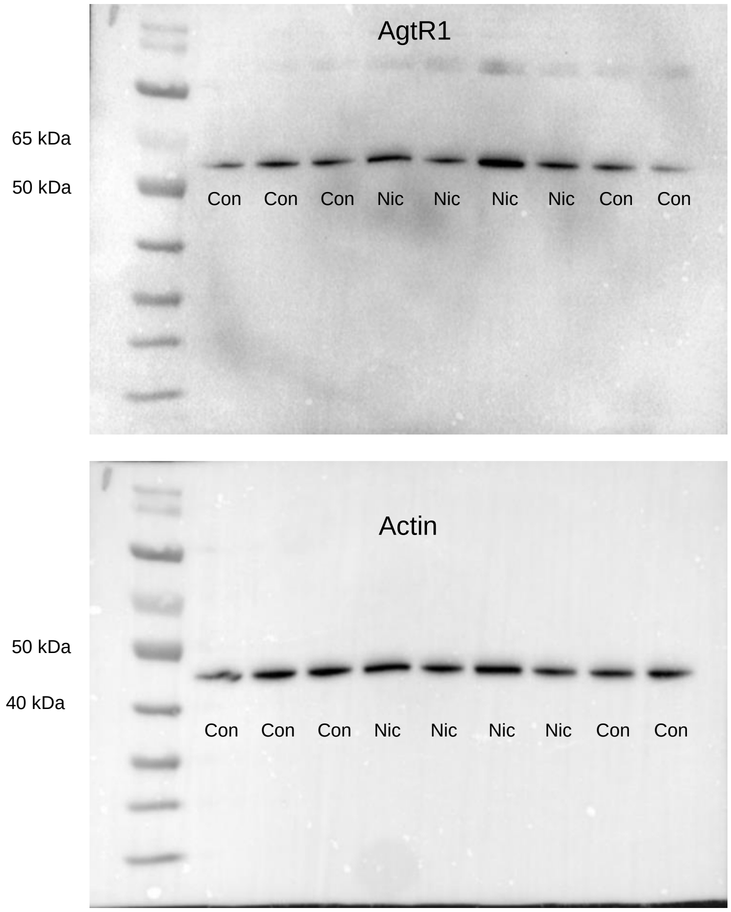

Figure S4. Immunoblots of PC12 cells used for analysis. Untouched blots show control (n=5) and nicotine (n=4) replicates. Actin was stained first, then the membrane was stripped and AgtR1 was stained second. AgtR1 was expressed relative to Actin (Figure 3C).

Figure S5.

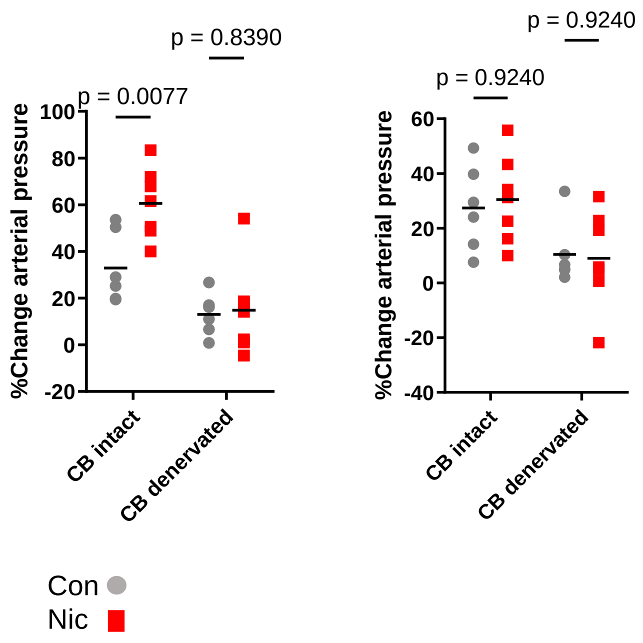

Figure S5. Carotid body reactivity to angiotensin is increased in perinatal nicotine-exposed pups compared to control. The percentage change in arterial pressure in response to angiotensin in vehicle (grey) and perinatal-nicotine exposed pups (red) before and following carotid body denervation $F_{1, 11}$=7.171). The percentage change in arterial pressure in response to NaCN in vehicle (grey) and perinatal-nicotine exposed pups (red) before and following carotid body denervation $F_{1, 11}$=0.6972). P-values for comparisons using Holm-Sidak post-hoc tests are inset in figure. n=6 per group

Figure S6.

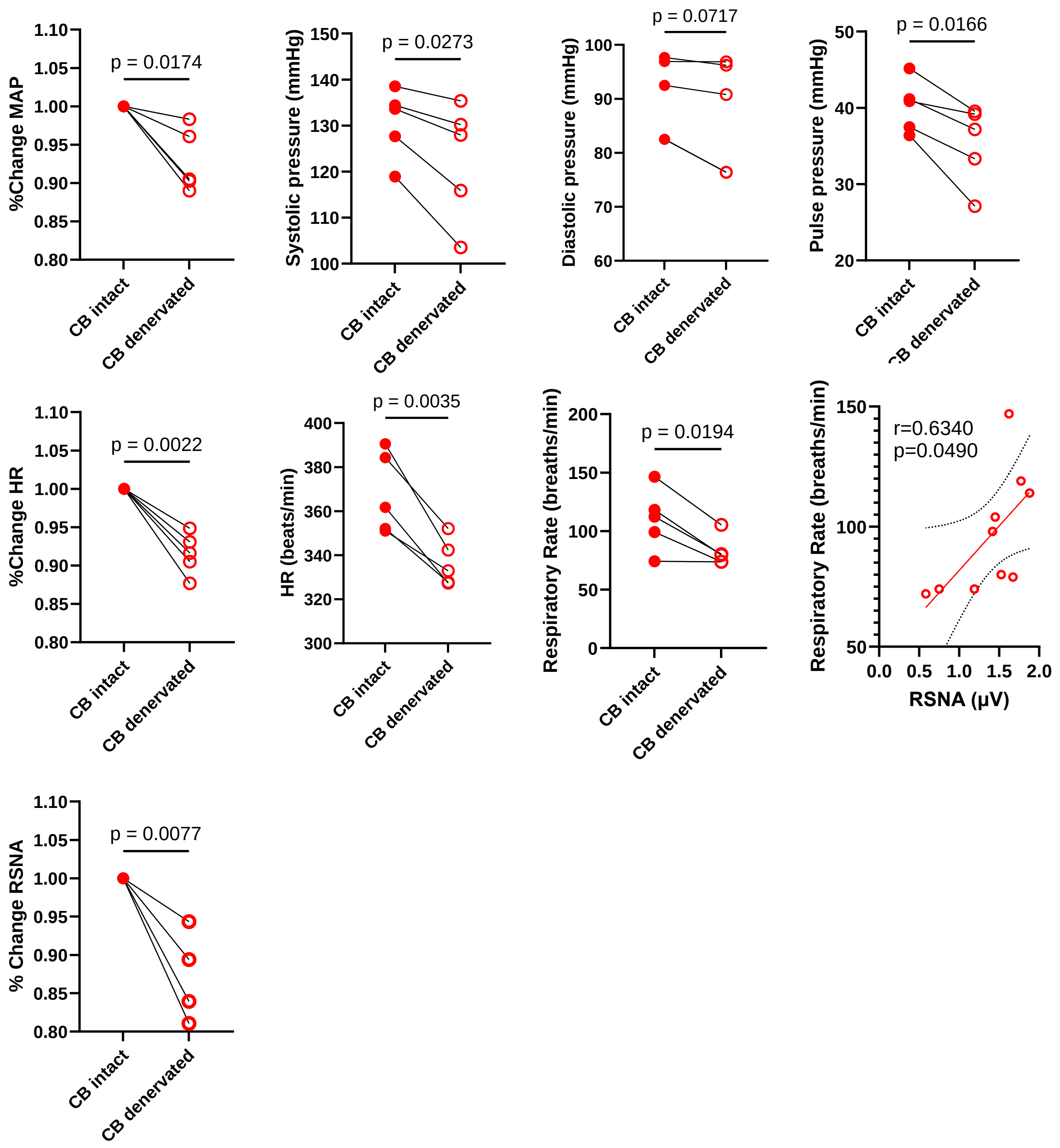

Figure S6. Perinatal nicotine-exposed rats have increased mean arterial pressure mediated by carotid body augmentation of sympathetic activity. Summary statistics in n=5 perinatal nicotine rats comparing the percent change of MAP(T_4=3.908), Systolic pressure (T_4=3.399) and Diastolic pressure (T_4=2.433) Pulse pressure (T_4=3.966), %Change HR (T_4=6.971) and HR (beats/min, T_4=6.180), Respiratory rate (obtained from pressure waveform filtered at 300Hz low pass filter, T_4=3.783), Correlation of respiratory rate and RSNA, Pearson Correlation, and %Change RSNA(T_4=4.956). n=5 per group.
