# Supplementary material for: Epigenetic Upregulation of Carotid Body Angiotensin Signaling Increases Blood Pressure
Source: Hypertension. 2024 Dec 5;82(2):293–305. doi: 10.1161/HYPERTENSIONAHA.124.23349 (PMC11732265; doi:10.1161/HYPERTENSIONAHA.124.23349)
Supplement: Supplementary file 5 [file hyp-82-293-s005.pdf]

* **ARRIVE Reporting Guidelines**
This study involves use of animal models:
Yes

Manuscripts reporting on animal research are expected to adhere to the ARRIVE guidelines. A completed ARRIVE reporting guideline checklist will be required prior to acceptance and the information should be clearly presented in the manuscript. The following shortened version of the checklist should be completed at this time; a completed ARRIVE checklist is required to be uploaded during revision submission.

Animals
Species, age, sex, strain, and sources of animals are described:
Yes

Randomization
Randomization and allocation concealment were performed and described:
Yes

Blinding
Blinding was performed and the process described:
Yes

Inclusions and Exclusions (a)
Specific criteria for inclusions and exclusions are specified:
N/A

Inclusions and Exclusions (b)
Criteria for inclusions and exclusion were set before the study:
N/A

Reporting of Excluded Animals
All animals excluded after the randomization are reported:
N/A

Statistical Methods
Statistical Methods are described:
Yes

Date completed: 11/12/2024 19:31:43
User pid: 908373
